# Musculoskeletal Inverse Kinematics Tool for Inertial Motion Capture Data Based on the Adaptive Unscented Kalman Smoother: An Implementation for OpenSim

**DOI:** 10.1007/s10439-025-03807-x

**Published:** 2025-07-28

**Authors:** Matti J. Kortelainen, Paavo Vartiainen, Alexander Beattie, Jere Lavikainen, Pasi A. Karjalainen

**Affiliations:** 1 Department of Technical Physics, University of Eastern Finland, P.O. Box 1627, 70211 Kuopio, Finland; 2 PSHVA Research Services, Kuopio University Hospital, Wellbeing Services County of North Savo, Kuopio, Finland

**Keywords:** Kalman filters, Motion estimation, Inertial sensors, Open source software

## Abstract

**Purpose:**

Conventional tools for human kinematics estimation presume that observations are subject to uncorrelated, zero-mean Gaussian noise, and they provide no estimate for the uncertainty of their solutions. This paper presents AUKSMIKT—a tool for whole-body kinematics estimation in the Bayesian framework to account for these shortcomings.

**Methods:**

We implemented AUKSMIKT as a C++ class that extends the OpenSim (v4.5) application programming interface. AUKSMIKT is based on the unscented Kalman filter combined with a run-time estimator of process and observation noises, and a fixed-lag Rauch-Tung-Striebel smoother. We tested the performance of AUKSMIKT using data from a public dataset consisting of both optical and inertial motion capture data recorded from overground walking subjects. We computed the mean absolute errors of estimated angular positions, velocities, and accelerations with respect to the gold standard optical motion capture estimates, and compared these metrics to those obtained from the least squares estimation-based tool native to OpenSim.

**Results:**

AUKSMIKT produced smaller errors than the native tool for the angular position of three joints (0.8–1.9%), the velocities of six joints (0.7-$-$7.6%), and the accelerations of seven joints (3.0–13.7%). AUKSMIKT produced larger errors in the angular positions of five joints (1.3–7.6%), and the velocities of three joints (4.4–8.3%).

**Conclusion:**

With respect to the optical motion capture solution, AUKSMIKT can estimate lower-body kinematics from inertial motion capture data with comparable or higher accuracy than the native OpenSim least squares estimator.

## Introduction

Human motion analysis provides valuable biomechanics information for physiotherapy, rehabilitation, and the design of exoskeletons and prostheses [1–4]. This information can be obtained with OpenSim—a widely used open source software for simulating and analyzing human kinematics and kinetics by modeling the human as an articulated multibody system [5]. A standard, complete workflow consists of multiple sequential processing stages, the outputs of which are used as inputs of the following stages. In short,

1. motion capture and measurement of associated signals are used to provide time series data of observations that indirectly describe the motion,
2. inverse kinematics provides time series of estimates for human body pose and its time derivatives,
3. inverse dynamics provides time series of estimates for overall moments of external and muscle forces acting on the body, and
4. neuromusculoskeletal modeling provides a time series of estimates for muscle activation levels and muscle force magnitudes [6].

It is evident that uncertainties in the outputs of these stages propagate and accumulate throughout this workflow. Especially the uncertainties of early stages amplify and can lead to large errors in the estimates of kinetics [7]. Despite this, the native inverse kinematics tools of OpenSim for optical and/or inertial motion capture data currently lack the ability to account for potentially time-variant uncertainties, as well as to quantify uncertainties in the output.

A promising solution for this problem is migrating the motion estimation workflow to the Bayesian framework. In general settings of probabilistic state estimation, Bayesian filters recursively fuse the available prior knowledge on the uncertainties of the state and observations in the estimation of the posterior distribution of the state of a dynamic system. Assuming the states and observations are normally distributed, the estimation may be carried out with (nonlinear) Kalman filters [8]—such as the extended Kalman filter (EKF) and the unscented Kalman filter (UKF).

Probabilistic state estimation in the context of human motion estimation has been explored in a number of studies. In [9], the EKF was combined with the fixed-interval Rauch–Tung–Striebel (RTS) smoother [10], which estimates the state of the system by utilizing both past and future observations [11], was used to reduce estimation errors of generalized coordinates in OpenSim based on optical motion capture data. In [12], the UKF algorithm and RTS smoother were used with a custom-made rigid-body model to estimate accelerations of body joints based on optical motion capture data. In [13], sagittal plane kinetics were estimated with a constrained EKF algorithm, which improved the estimation of ground reaction forces and moments. In [14], the UKF algorithm was used for upper limb pose estimation with magnetometer-free inertial measurement units (IMUs). The authors of [15] compared the EKF against the UKF in estimating angular positions of robot arm joints from IMU data and found that the UKF performs slightly better.

Although the (marker-based) optical method is often considered the gold standard in motion capture [16–18], the use of IMUs is an appealing alternative since their more straightforward setup and less limited field of view (FOV) facilitate motion capture outside dedicated motion laboratories. To our knowledge, we present in this paper the first probabilistic inverse kinematics tool for inertial motion capture data in OpenSim. This open source tool, entitled ‘Adaptive Unscented Kalman Smoothing Musculoskeletal Inverse Kinematics Tool’ (AUKSMIKT), is based on the UKF algorithm combined with run-time noise statistics estimation and fixed-lag RTS smoothing, and extends the application programming interface (API) of the OpenSim software. We benchmark the ability of this tool to estimate the time series of human body kinematics with a public inertial motion capture dataset representing human walking against optical motion capture, and compare this performance to the native inverse kinematics tool of OpenSim. In addition, we measure the throughput of this tool, and discuss potential improvements.

## Materials and Methods

In order to account for the uncertainty of observations in the estimation of kinematics, we developed AUKSMIKT—a probabilistic inverse kinematics tool based on the UKF algorithm. In Sect. 2.1, we present the general formulation of the adaptive UKF algorithm and the RTS smoothing algorithm. In Sect. 2.2, we present these algorithms in the context of kinematics estimation. In Sect. 2.3, we describe the performance tests carried out for AUKSMIKT.

### Adaptive Unscented Kalman Filtering and Smoothing

In this section, we present the adaptive UKF algorithm with RTS smoothing. Algorithm 1 presents a high-level outline of the algorithm as a whole. For details, Algorithm 2 presents the adaptive UKF update loop, and Algorithm 3 presents the backwards smoothing feature, i.e., the RTS smoother of AUKSMIKT.

#### Unscented Kalman Filter

Let $\textbf{x}_{(k)}\in \mathbb {R}^{n}$, i.e., an unknown *n*-dimensional state vector of real numbers, that describes a dynamic system which yields an *m*-dimensional vector of observations $\textbf{y}_{(k)}\in \mathbb {R}^{m}$ for each time frame $k = 0, 1, \dots , K$. Assuming a Markovian state space model and conditionally independent observations, these states and observations are related through (nonlinear) process and observation models as [11]

1 $$\begin{aligned} \textbf{x}_{(k)}&= \textbf{f}\left( \textbf{x}_{(k-1)}\right) + \textbf{w}_{(k)}, \end{aligned}$$

2 $$\begin{aligned} \textbf{y}_{(k)}&= \textbf{h}\left( \textbf{x}_{(k)}\right) + \textbf{v}_{(k)}, \end{aligned}$$

where $\textbf{f}(\cdot )$ is the state transition function, $\textbf{h}(\cdot )$ is the observation function, $\textbf{w}_{(k)}\in \mathbb {R}^{n}$ is the process noise, and $\textbf{v}_{(k)}\in \mathbb {R}^{m}$ is the observation noise.

We further assume that vectors $\textbf{x}_{(k)}$ belong to multivariate normal distributions with means $\bar{\textbf{x}}_{(k)}$ and covariance matrices $\textbf{P}_{(k)}$, and that $\textbf{w}_{(k)}$ and $\textbf{v}_{(k)}$ belong to multivariate normal distributions with means $\bar{\textbf{w}}_{(k)}$ and $\bar{\textbf{v}}_{(k)}$, respectively, and covariance matrices $\textbf{Q}_{(k)}$ and $\textbf{R}_{(k)}$, respectively. Under these circumstances, the distributions of $\textbf{x}_{(k)}$ can be estimated with the unscented Kalman filter (UKF) algorithm [19]. A general, so-called non-augmented UKF algorithm for state estimation is jointly presented in Algorithms 1 and 2. In Algorithm 2, the function $\textit{choleskyDecomp}(\cdot )$ computes the Cholesky factor $\textbf{L}$ of a given matrix $\textbf{M}$ such that $\textbf{M} = \textbf{L}\textbf{L}^{T}$, and the notation $\textbf{M}_{\left[ :,\,i\right] }$ refers to selecting column *i* from matrix $\textbf{M}$. In summary, we recursively estimate the posterior density of $\textbf{x}_{(k)}$ by

1. sampling the posterior density of step $k-1$ in the proximity of $\bar{\textbf{x}}_{(k-1)}$ (Algorithm 2, lines 1–2),
2. propagating these so-called sigma points through the (nonlinear) process model (Algorithm 2, line 3),
3. predicting the density of step *k* (Algorithm 2, lines 4–6),
4. sampling this prior density of step *k* (Algorithm 2, lines 8–9),
5. propagating these re-sampled sigma points through the (nonlinear) observation model (Algorithm 2, line 10),
6. computing the weighed mean and covariance of these propagated sigma points (Algorithm 2, lines 11–13), and
7. updating the density of step *k* using these statistics and observations $\textbf{y}_{(k)}$ (Algorithm 2, lines 14–17).

In general, UKF has three adjustable hyperparameters $\alpha$, $\beta$, and $\kappa$, which control how far away from the mean the sigma points are collected and how these sigma points are weighed (weights $W_{(i)}^{(m)}$ and $W_{(i)}^{(c)}$) in the estimation of moments of the state.

#### Estimation of Noise Statistics

The performance of the UKF depends significantly on the accuracy of the noise statistics $\bar{\textbf{w}}_{(k)}$, $\textbf{Q}_{(k)}$, $\bar{\textbf{v}}_{(k)}$, and $\textbf{R}_{(k)}$ used in the filtering [20]. Since these statistics are rarely known a priori, their ‘correct’ values need to be estimated with respect to the observed data and adopted models. In covariance matching, the aim is to update the matrices $\textbf{Q}_{(k)}$ and $\textbf{R}_{(k)}$ such that they become more consistent with empirical residual vectors $\textbf{y}_{(k)} - \textbf{h}\left( \bar{\textbf{x}}_{(k)}\right)$ [21]. Two common update methods for covariance matching are the forgetting factor method and the moving window method [22]. In the forgetting factor method, a new estimate is obtained as a weighted sum of the covariance matrix that best matches the residuals during time step *k* and the previous covariance matrix, with weights $\eta$ and $1-\eta$, respectively. Alternatively, one can obtain estimates for $\bar{\textbf{w}}_{(k)}$, $\textbf{Q}_{(k)}$, $\bar{\textbf{v}}_{(k)}$, and $\textbf{R}_{(k)}$ by maximizing the posterior density $p\left( {\textbf{X}}_{(k)}, \bar{\textbf{w}}, \textbf{Q}, \bar{\textbf{v}}, \textbf{R} \; | \, \textbf{Y}_{(k)} \right)$, where $\textbf{X}_{(k)} = \left\{ \textbf{x}_{(\ell )}\right\} _{\ell = 0}^{k}$ and $\textbf{Y}_{(k)} = \left\{ \textbf{y}_{(\ell )}\right\} _{\ell = 0}^{k}$ [23, 24]. Lines 18–21 in Algorithm 2 present a method for estimating and updating the noise statistics based on this maximum a posteriori approach combined with the forgetting factor method.

#### Fixed-Lag Unscented Kalman Smoothing

Due to the infinite impulse response (IIR) of the UKF, the estimated densities of elements of the state $\textbf{x}_{(k)}$ may be subject to a delay or a lag with respect to the corresponding observations $\textbf{y}_{(k)}$ [25]. To counteract this delay, one can apply unscented Rauch-Tung-Striebel (RTS) smoothing [26] by updating the densities with the estimates from time frames $K, K-1, \dots , k$ and going recursively backwards in time—analogous to forward-backward filtering with IIR causal filters. Since this type of fixed-interval smoothing would prohibit potential real-time use cases, we approximate the unscented RTS smoothing by truncating the impulse response of the backward smoothing pass to *L* time frames, thus implementing a fixed-lag smoother. This fixed-lag RTS smoothing is presented in Algorithm 3.

### UKF-Based Estimation of Inverse Kinematics

To solve inverse kinematics of musculoskeletal (MS) models within the UKF framework, we considered the state vectors $\textbf{x}_{(k)}$ as concatenated vectors of generalized (mutually independent) rotational coordinates and their time derivatives $\textbf{q}_{(k)}^{(d)},\,d = 0,1,\dots ,D$, i.e., positions (angles; $d = 0$), velocities ($d = 1$), accelerations ($d = 2$), etc., of body joints

$$ \textbf{x}_{(k)} = \begin{bmatrix}\left[ \textbf{q}_{(k)}^{(0)}\right] ^{T}&\left[ \textbf{q}_{(k)}^{(1)}\right] ^{T}&\dots&\left[ \textbf{q}_{(k)}^{(D)}\right] ^{T}\end{bmatrix}^{T}, $$

where $\textbf{q}_{(k)}^{(d)} \in \mathbb {R}^{n_f} \, \forall d$, and $n_f = n / (D+1)$ is the number of generalized coordinates.

We assume that the angular positions may be presented as infinitely differentiable functions; thus we adopted the conventional model presented in [27] as the process model (1). In this model, each element *i* of time derivatives $\textbf{q}^{(D+1)}(t)$, where *t* is time, is assumed to be an independent, normally distributed random variable with the mean of zero and the variance $\sigma _{i}^{2}$. In this case, the state transition function $\textbf{f}(\cdot )$ is a matrix–vector product, with the matrix containing the terms of corresponding Taylor series expansions. Thus

3 $$\begin{aligned} \begin{aligned} \textbf{f}\left( \textbf{x}_{(k)}\right)&= \begin{bmatrix}\textbf{I} &  \Delta t\cdot \textbf{I} &  \dots &  \frac{\left( \Delta t\right) ^{D}}{D!}\cdot \textbf{I} \\ \textbf{0} &  \textbf{I} &  \dots &  \frac{\left( \Delta t\right) ^{D-1}}{(D-1)!}\cdot \textbf{I} \\ \vdots &  \vdots &  \ddots &  \vdots \\ \textbf{0} &  \textbf{0} &  \dots &  \textbf{I} \\ \end{bmatrix}\begin{bmatrix}\textbf{q}_{(k)}^{(0)} \\ \textbf{q}_{(k)}^{(1)} \\ \vdots \\ \textbf{q}_{(k)}^{(D)}\end{bmatrix} \\&= \textbf{F}\textbf{x}_{(k)}, \\ \end{aligned} \end{aligned}$$

where $\Delta t$ is the time interval between consecutive observations, $\textbf{I}$ is an identity matrix of size $n_f \times n_f$, and $\textbf{0}$ is a matrix of zeros of size $n_f \times n_f$. Correspondingly, the process noise $\textbf{w}_{(k)}$ is modeled as [27]

4 $$\begin{aligned} \begin{aligned} \textbf{w}_{(k)} =&\int _{0}^{\Delta t} \textbf{q}^{(D+1)}\left( t + \Delta t - \theta \right) \; \otimes \\&\left[ \begin{array}{cccc} c_{0}\frac{\left( \theta \right) ^{D}}{D!}&c_{1}\frac{\left( \theta \right) ^{D-1}}{\left( D-1\right) !}&\dots&c_{D}\end{array}\right] ^{T} \,\!\textrm{d}\theta , \end{aligned} \end{aligned}$$

where $c_i \in \mathbb {R},\, i=0,1,\dots ,D$ are optional scaling parameters—set equal to ones by default—and $\otimes$ denotes the Kronecker product. Accordingly, the process noise covariance $\textbf{Q}_{(k)}$ is

5 $$\begin{aligned} \begin{aligned} \textbf{Q}_{(k)} =&\begin{aligned} \int _{0}^{\Delta t}&\left[ \begin{array}{cccc} c_{0}\frac{\left( \theta \right) ^{D}}{D!}&c_{1}\frac{\left( \theta \right) ^{D-1}}{\left( D-1\right) !}&\dots&c_{D}\end{array}\right] ^{T}\cdot&\\&\left[ \begin{array}{cccc} c_{0}\frac{\left( \theta \right) ^{D}}{D!}&c_{1}\frac{\left( \theta \right) ^{D-1}}{\left( D-1\right) !}&\dots&c_{D}\end{array}\right] \,\!\textrm{d}\theta \\&\otimes \textit{dia}\left( \mathbf{\Sigma }\right) \end{aligned} \\ =&\left[ \begin{array}{cccc} Q_{0,0}\cdot \textit{dia}(\mathbf{\Sigma }) &  Q_{0,1}\cdot \textit{dia}(\mathbf{\Sigma }) &  \dots &  Q_{0,D}\cdot \textit{dia}(\mathbf{\Sigma }) \\ Q_{1,0}\cdot \textit{dia}(\mathbf{\Sigma }) &  Q_{1,1}\cdot \textit{dia}(\mathbf{\Sigma }) &  \dots &  Q_{1,D}\cdot \textit{dia}(\mathbf{\Sigma }) \\ \vdots &  \vdots &  \ddots &  \vdots \\ Q_{D,0}\cdot \textit{dia}(\mathbf{\Sigma }) &  Q_{D,1}\cdot \textit{dia}(\mathbf{\Sigma }) &  \dots &  Q_{D,D}\cdot \textit{dia}(\mathbf{\Sigma }) \end{array}\right] , \end{aligned} \end{aligned}$$

where $$\mathbf{\Sigma } = \begin{bmatrix}\sigma _{1}^{2}&\sigma _{2}^{2}&\dots&\sigma _{n_f}^{2} \end{bmatrix}^{T}$$, function $\textit{dia}(\cdot )$ creates a diagonal matrix from the input vector, and the coefficients $Q_{i,j}$ are given by [27]

6 $$\begin{aligned} \begin{aligned} Q_{i,j} =&\frac{c_{i}c_{j}(\Delta t)^{2D + 1 - (i + j)}}{(D - i)!(D - j)!\left( 2D + 1 - (i + j)\right) }, \\  &i,j = 0,1,\dots ,D. \end{aligned} \end{aligned}$$

The choice of $\sigma _{i}^{2}$ values could be based on the power spectral analysis of the kinematics [27]; however, since this information may not be available, and the power spectra of kinematics may change over time, the values of $\sigma _{i}^{2}$ need to be updated adaptively (Sect. 2.1.2).

**Figure:**
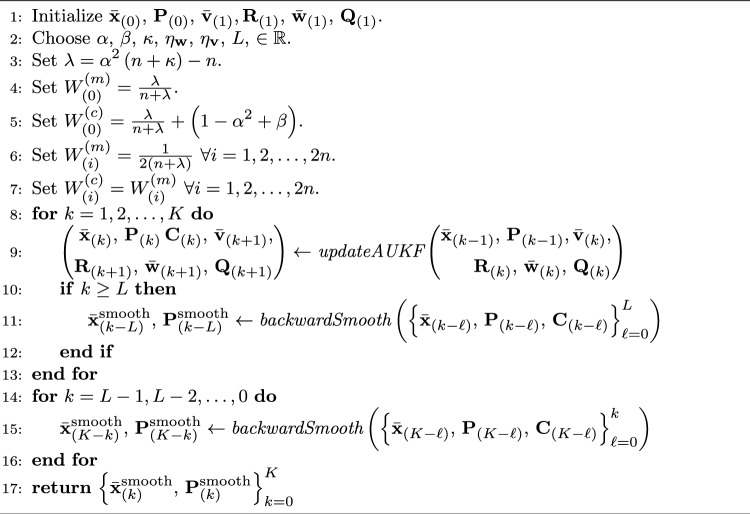
Algorithm 1. Adaptive Unscented Kalman Filter and Smoother for state estimation

We note that since the process model (1) is linear with respect to the state $\textbf{x}_{(k)}$ with the above-mentioned choices, we can replace the prediction steps of UKF with corresponding linear variants of Kalman filter and RTS smoother [10]. In addition, the means $\bar{\textbf{w}}_{(k)}$ will be zero with the above-mentioned choices. Thus, lines 1–7 in Algorithm 2 can be reduced to linear transformations [10]

7 $$\begin{aligned} \bar{\textbf{x}}_{(k)}^{-}&= \textbf{F}\bar{\textbf{x}}_{(k-1)} \end{aligned}$$

8 $$\begin{aligned} \textbf{P}_{(k)}^{-}&= \textbf{F}\textbf{P}_{(k-1)}\textbf{F}^{T} + \textbf{Q}_{(k)} . \end{aligned}$$

9 $$\begin{aligned} \textbf{C}_{(k)}&= \textbf{P}_{(k-1)}\textbf{F}^{T}. \end{aligned}$$

To ensure the updated covariance matrices $\textbf{Q}_{(k+1)}$ remain consistent with the adopted model (5), lines 18–19 in Algorithm 2 are replaced with

10 $$\begin{aligned} \tilde{\textbf{Q}}&= \begin{bmatrix}\bar{\textbf{x}}_{(k)} - \textbf{F}\bar{\textbf{x}}_{(k-1)}\end{bmatrix} \cdot \begin{bmatrix}\bar{\textbf{x}}_{(k)} - \textbf{F}\bar{\textbf{x}}_{(k-1)}\end{bmatrix}^{T} \end{aligned}$$

11 $$\begin{aligned} \tilde{\sigma }_{i}^{2}&= \frac{\tilde{\textbf{Q}}_{(D-1)n_f + i,\, (D-1)n_f + i}}{Q_{D,D}}, \; i = 1,2,\dots n_f \end{aligned}$$

12 $$\begin{aligned} \sigma _{i}^{2}&= \eta _{\textbf{w}}\tilde{\sigma }_{i}^{2} + \left( 1 - \eta _{\textbf{w}}\right) \sigma _{i}^{2}, \; i = 1,2,\dots n_f \end{aligned}$$

13 $$\begin{aligned} \mathbf{\Sigma }&= \begin{bmatrix}\sigma _{1}^{2}&\sigma _{2}^{2}&\dots&\sigma _{n_f}^{2} \end{bmatrix}^{T} \end{aligned}$$

**Figure:**
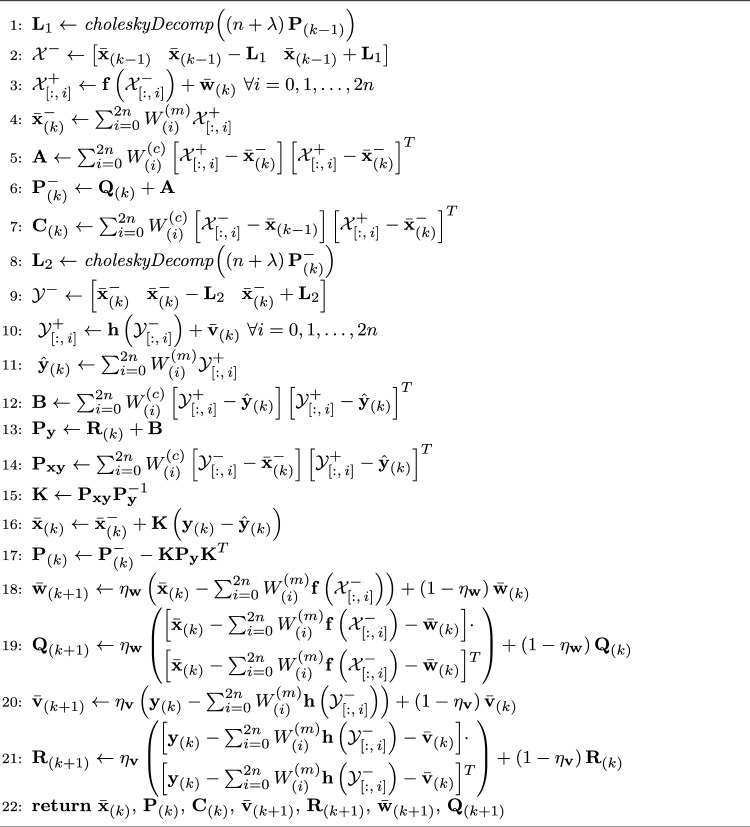
Algorithm 2. Function *updateAUKF* $\left({{\bar{x}}}_{(k-1)},\, \textbf{P}_{(k-1)}, \, {{\bar{\textbf{v}}}}_{(k)}, \, \textbf{R}_{(\bar{k})}, \, {{\bar{\textbf{w}}}}_{(k)}, \, \textbf{Q}_{(k)}\right)$ [19, 23]

14 $$\begin{aligned} \textbf{Q}_{(k+1)}&= \begin{bmatrix}Q_{0,0}\cdot \textit{dia}(\mathbf{\Sigma }) &  Q_{0,1}\cdot \textit{dia}(\mathbf{\Sigma }) &  \dots &  Q_{0,D}\cdot \textit{dia}(\mathbf{\Sigma }) \\ Q_{1,0}\cdot \textit{dia}(\mathbf{\Sigma }) &  Q_{1,1}\cdot \textit{dia}(\mathbf{\Sigma }) &  \dots &  Q_{1,D}\cdot \textit{dia}(\mathbf{\Sigma }) \\ \vdots &  \vdots &  \ddots &  \vdots \\ Q_{D,0}\cdot \textit{dia}(\mathbf{\Sigma }) &  Q_{D,1}\cdot \textit{dia}(\mathbf{\Sigma }) &  \dots &  Q_{D,D}\cdot \textit{dia}(\mathbf{\Sigma })\end{bmatrix} , \end{aligned}$$

where coefficients $Q_{i,j}$ are the same as in (6).

In our case, the observations $\textbf{y}_{(k)}$ are orientations obtained from inertial measurement units (IMUs) attached to the human body, which can be expressed either as unit quaternions or Euler angles. We used both expressions in our implementation, depending on the purpose. Since the uncertainties of the IMUs were given with respect to Euler (or Tait-Bryan, to be exact [28]) angles, the initial observation noise covariance matrix was constructed as

**Figure:**
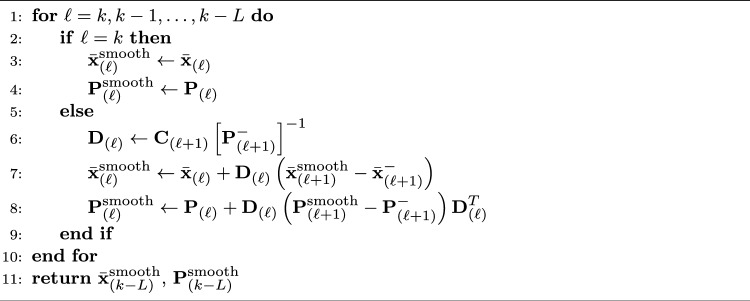
Algorithm 3. Function *backwardSmooth* $\left(\left\{\bar{\textbf{x}}_{(k - \ell)}, \, \textbf{P}_{(k - \ell)}, \, \textbf{C}_{(k-\ell)}\right\}_{\ell = 0}^{L}\right)$ [26]

15 $$\begin{aligned} \textbf{R}_{(1)} = dia \left( \begin{bmatrix}\nu _{x,\,1}^{2}&\nu _{y,\,1}^{2}&\nu _{z,\,1}^{2}&\dots&\nu _{z,\,m_{\text {E}}}^{2}\end{bmatrix}\right) , \end{aligned}$$

where $\nu _{x,\,i}^{2}$, $\nu _{y,\,i}^{2}$ and $\nu _{z,\,i}^{2}$ are the squares of the root-mean-square (RMS) errors of IMU *i* about the *x*-, *y*- and *z*-axes in the ground coordinate system, respectively, when the orientations are expressed in Euler angles, $m_{\text {E}} = m/3$ is the number of IMUs, and the function $\textit{dia}(\cdot )$ creates a diagonal matrix from the input vector. Appropriately, the results of the orientation subtractions $\mathcal {Y}_{\left[ :,\,i\right] }^{+} - \hat{\textbf{y}}_{(k)}$ and $\textbf{y}_{(k)} - \hat{\textbf{y}}_{(k)}$ were converted from unit quaternions to Euler angles before computing the covariances $\textbf{P}_{\textbf{y}}$ and $\textbf{P}_{\textbf{xy}}$ (Algorithm 2, lines 12–14) and the posterior mean $\bar{\textbf{x}}_{(k)}$ (Algorithm 2, line 16), respectively. On the other hand, to avoid numerical ambiguity, the orientation subtractions themselves were computed using unit quaternions. That is, for an individual IMU *i*, the subtraction of two orientations $\textbf{a}_{(i)}$ and $\textbf{b}_{(i)}$ expressed in Euler angles corresponds to the Hamilton product of two unit quaternions as [29]

$$ \textbf{a}_{(i)}^{(\text {E})} - \textbf{b}_{(i)}^{(\text {E})}\; \simeq \; \textbf{b}_{(i)}^{(\text {Q})}\otimes _{\text {H}}\overline{\left( \textbf{a}_{(i)}^{(\text {Q})}\right) }, $$

where superscript $(\text {E})$ refers to an orientation expressed in Euler angles, superscript (Q) refers to an orientation expressed in quaternions, $\overline{\left( \cdot \right) }$ is the conjugation operation, and $\otimes _{\text {H}}$ is an operator for the Hamilton product. Further, the orientations $\hat{\textbf{y}}_{(k)}$ (Algorithm 2, line 11) were computed as weighted average quaternions using the method presented in [30].

The updating of the covariance matrix $\textbf{R}_{(k+1)}$ was carried out as outlined in lines 20–21 in Algorithm 2, with a modification that the off-diagonal elements corresponding to the cross-covariances between different IMUs were always set to zero. That is, we assume the IMUs are mutually independent, but allow the observations with respect to different coordinate axes to be correlated within the same IMU.

The initial state mean $\bar{\textbf{x}}_{(0)}$ was constructed as the least squares (LS) solution of the corresponding observation $\textbf{y}_{(0)}$ (see Sect. 2.3.2). The initial process and observation noise means $\bar{\textbf{w}}_{(1)}$ and $\bar{\textbf{v}}_{(1)}$, respectively, were initialized as zeros, and the process noise covariance $\textbf{Q}_{(1)}$ was constructed using the same value for all variances $\sigma _{i}^{2}$. The initial state covariance $\textbf{P}_{(0)}$ was set equal to $\textbf{Q}_{(1)}$.

The source code of our software implementation is available on GitHub (https://github.com/Sandmaenchen/opensim-core-public/tree/ukf-uks-tools), and is released under the Apache License 2.0. A detailed description of this implementation is provided in Appendix 5.

### Testing of AUKSMIKT

In this section, we present the benchmark tests evaluating the ability of AUKSMIKT to estimate the time series of human body kinematics, as well as its throughput.

#### Test Data

For testing, we used the Kuopio Gait dataset [31, 32] which consisted of both optical and inertial motion capture data subsets from 51 individuals performing multiple gait trials at three different walking speeds. Seven IMUs had been placed on the subjects’ pelvis, thighs, shanks and feet and 42 reflective markers had been attached to the subjects’ skin following the protocol described by [32]. The IMUs had been recorded at a sampling rate of 100 Hz. Of the available data subsets, we selected the ones where the subjects were walking in the *left* direction at a *comfortable* walking speed [32].

The base MS model used in these tests was a full-body model [33], the arms of which had been removed and the knee structure replaced with one capable of adduction [34]. This model had 20 independent rotational coordinates. Subject-specific MS models were created by scaling the body segments of the base MS model using a combination of manual anthropometric measurements and experimental marker locations recorded during the calibration pose (N-pose). Further information about these procedures is provided in Appendix 6.

#### Inverse Kinematics

Before computing the IMU-driven LS solution and the adaptive UKS (AUKS) solution, we reoriented the virtual IMUs on the subject-specific MS model such that their orientations with respect to their parent body segments matched the calibration pose of the MS model at the start of each gait trial. This minimizes any bias caused by inter-trial IMU signal drift. We set the limits of inequality constraints (property *clamped* of the coordinates of the MS model) to $\left[ -170,\, 170\right]$ (degrees) before calculating the IMU-driven solutions. This was done due to the observed systematic offsets in estimated angular positions between the two modalities, which would have led to applying the constraints only on the IMU-driven solutions if a smaller window of permitted values was used.

The native OpenSim inverse kinematics tool for optical motion capture data was used to compute solutions $\hat{\textbf{q}}_{(k)}^{(0),\text {optical}}$ by frame-wise minimizing the LS distance between the observed marker locations and the locations predicted by the MS model for a given state. The durations of these LS solutions varied between 1.5 s and 4.0 s (mean: 2.83 s, standard deviation: 0.75 s). These solutions $\hat{\textbf{q}}_{(k)}^{(0),\text {optical}}$ were forward-backward (zero phase shift) low-pass filtered (LPF) with a 3rd-order Butterworth filter with a cutoff frequency of 6.0 Hz [35]. The LPF LS solution $\bar{\textbf{q}}_{(k)}^{(0),\text {optical}}$ for angular positions was sampled from a 5th-degree generalized cross-validation spline [36] fitted to the LPF signal. The 1st-order and 2nd-order time derivatives of the spline were sampled to yield angular velocities $\bar{\textbf{q}}_{(k)}^{(1),\text {optical}}$ and angular accelerations $\bar{\textbf{q}}_{(k)}^{(2),\text {optical}}$, respectively. These LPF LS solutions form the ground truths utilized in subsequent statistical analyses. Further information about optical motion capture solutions is given in Appendix 7.

The native OpenSim inverse kinematics tool for IMU data computes LS solutions $\hat{\textbf{q}}_{(k)}^{(0), \text {IMU}}$ frame-wise by minimizing the sum of squared differences between the orientations of virtual IMUs in the model and the observed orientations of corresponding physical IMUs [37]. These solutions $\hat{\textbf{q}}_{(k)}^{(0), \text {IMU}}$ were processed similar to $\hat{\textbf{q}}_{(k)}^{(0), \text {optical}}$ to obtain LPF LS solutions $\bar{\textbf{q}}_{(k)}^{(0),\text {IMU}}$, $\bar{\textbf{q}}_{(k)}^{(1),\text {IMU}}$, and $\bar{\textbf{q}}_{(k)}^{(2),\text {IMU}}$.

For AUKS solutions, we set the highest order of the time derivatives *D* to 2, lag length *L* to 10, scalar values $c_i$ to ones, and the forgetting factors to $\eta _{\textbf{w}} = 0.1$ and $\eta _{\textbf{v}} = 0.001$. The lower and upper limits for individual $\sigma _{i}$ parameters (see Appendix 5 for explanation) were set to $\sigma _{\text {min}}^{2} = 2^{10}$ and $\sigma _{\text {max}}^{2} = 3.0 \cdot 10^{7}$. The common UKF hyperparameters were set to $\alpha = 1.0$, $\beta = 2$, and $\kappa = 3 - n$. The value for $\eta _{\textbf{w}}$ was selected based on a grid search using a 40-s IMU dataset from a gait trial from Ref. [38], where we sought the minimum of the mean square error of the AUKS solutions $\bar{\textbf{x}}_{(k)}$ with respect to the corresponding LS solutions (no optical motion capture data were available). A subsequent grid search for $\eta _{\textbf{v}}$ suggested that values greater than $\eta _{\textbf{v}} = 0.0005$ lead to diverging solutions; however, since doubling this value did not cause divergences with the trials from the Kuopio Gait dataset, we used the value $\eta _{\textbf{v}} = 0.001$ instead.

#### Statistical Tests

Our initial screening of a subset of IMU-driven LS solutions in the OpenSim graphical user interface revealed the presence of significant artifacts in the IMU data for several test subjects; for example, the gait cycle could include an unusually large hip adduction that was not present in the optical motion capture data. Therefore, we selected the gait trials of only 13 subjects for whom the IMU-driven LS solutions were artifact-free based on visual inspection of two randomly chosen gait trials. These subjects were ‘01’, ‘03’, ‘04’, ‘05’, ‘06’, ‘08’, ‘09’, ‘12’, ‘13’, ‘15’, ‘18’, ‘40’, and ‘41’. There were five trials for which the IMU-driven LS solution failed to converge, and subject ‘03’ had two trials whose IMU data could not be processed; therefore, the total number of gait trials used in statistical tests was 123.

Considering the optical motion capture LS solutions $\bar{\textbf{q}}_{(k)}^{(d),\text {optical}},\, d=0,1,2,$ as the ground truth, we computed mean absolute errors (MAEs) over the gait trial for 12 rotational coordinates for each of the 123 gait trials and for both LS solutions $\bar{\textbf{q}}_{(k)}^{(d),\text {IMU}},\, d=0,1,2,$ and AUKS solutions $\bar{\textbf{x}}_{(k)}^{\text {smooth}}$. We excluded from the analysis the three lumbar coordinates and the two metatarsophalangeal coordinates due to the absence of IMUs (poor observability), and the three pelvis rotational coordinates due to different heading orientations of the pelvis between the optical and inertial motion capture data subsets. For each trial, calculation of MAE was carried out over the time window in which both optical and inertial motion capture solutions were present. To evaluate statistical significances of differences between the LS and AUKS solutions, we computed related-samples *t*-tests on the computed MAEs using the *Stats* module of Python library SciPy (v1.14.0). We also computed the 95% confidence intervals for the differences of the MAEs. Having a *p*-value below 0.05 was considered statistically significant.

#### Throughput Test

We tested the throughput of AUKSMIKT by processing the IMU data from a gait trial with a duration of 10.0 s for 5 consecutive times, and noting for each run the elapsed time between the beginning of the filtering loop (Algorithm 1, line 8) until the last frame was processed by the smoothing (Algorithm 1, line 16) by inspecting the log file written by the tool. The number of frames processed (i.e., 1000) was divided by this elapsed time to get the throughput as the number of frames processed per second. We repeated this procedure with different numbers of threads used by the thread pool.

This test was carried out on a Dell Latitude 5430 laptop computer with a 12th generation Intel Core i5-1235U CPU with 10 cores running at 1300 MHz. The computer was running Ubuntu Desktop (version 22.04.5) as the operating system. Calls to the tool were made through the C++ interface of OpenSim. The laptop computer was rebooted before the test, and no programs other than the terminal were launched before or during the test.

## Results

**Fig. 1:**
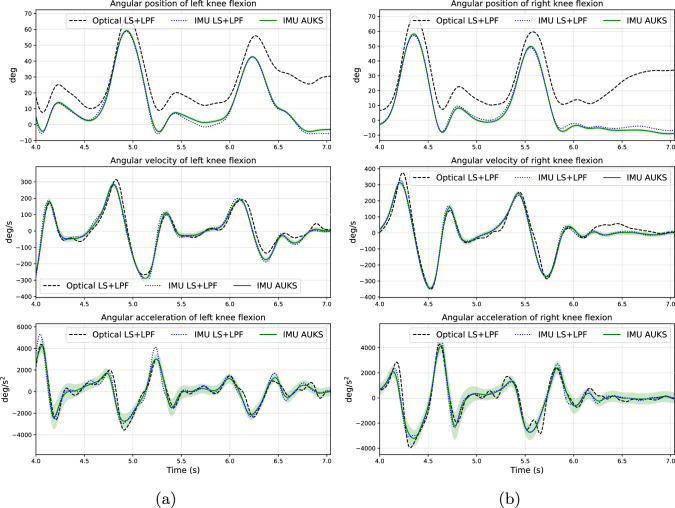
Example of kinematics of the knee flexion obtained as low-pass filtered (LPF) least squares (LS) solution based on optical motion capture data, as LPF LS solution based on IMU data, and as adaptive unscented Kalman smoothing (AUKS) solution based on IMU data, for the left leg (a) and the right leg (b). The green solid lines represent the estimated mean $\bar{\textbf{x}}_{(k)}^{\text {smooth}}$, and the pale green shaded regions represent the limits of mean ± 2 standard deviations — obtained from the estimated covariance $\textbf{P}_{(k)}^{\text {smooth}}$.

**Fig. 2:**
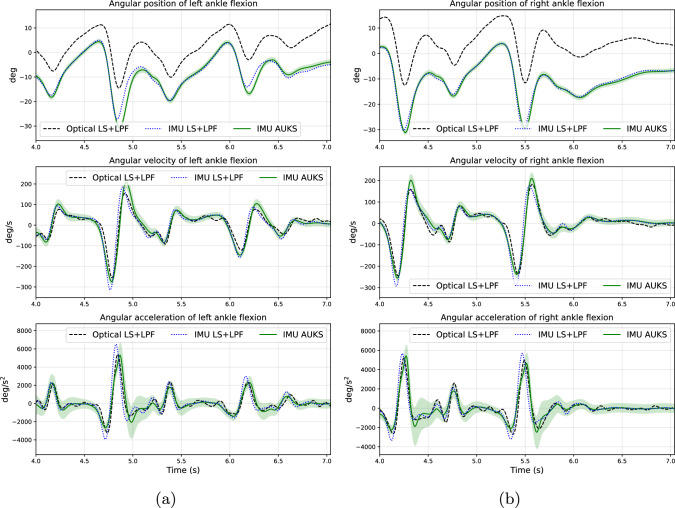
Example of kinematics of the ankle flexion obtained as low-pass filtered (LPF) least squares (LS) solution based on optical motion capture data, as LPF LS solution based on IMU data, and as adaptive unscented Kalman smoothing (AUKS) solution based on IMU data, for the left leg (**a**) and the right leg (**b**). The green solid lines represent the estimated mean $\bar{\textbf{x}}_{(k)}^{\text {smooth}}$, and the pale green shaded regions represent the limits of mean ± 2 standard deviations — obtained from the estimated covariance $\textbf{P}_{(k)}^{\text {smooth}}$.

Table 1 presents the computed MAEs for angular positions, velocities and accelerations of each evaluated coordinate. Figure 1 and Figure 2 present the optical motion capture LS solution, IMU-driven LS solution, and IMU-driven AUKS solution for kinematics of knee flexion and ankle flexion, respectively, for one gait trial of subject ‘09’.

For angular positions, the AUKS solutions had lower MAEs ($p < 0.05$) than the LS solutions in right hip flexion, right hip adduction, and left knee flexion, and higher MAEs in right hip rotation, left hip rotation, left knee adduction, right ankle flexion, and left ankle flexion.

For angular velocities, the AUKS solutions had lower MAEs ($p < 0.05$) than the LS solutions in left hip adduction, right knee flexion, left knee flexion, and left ankle flexion, and higher MAEs in right hip rotation, left knee adduction, and right ankle flexion.

For angular accelerations, the AUKS solutions had lower MAEs ($p < 0.05$) than the LS solutions in right hip flexion, right hip adduction, left hip adduction, left hip rotation, left knee flexion, right knee adduction, and left ankle flexion.

The results of the throughput test are presented in Table 2. The throughput improved by 68% by increasing the number of threads from one to three, and by 168% by increasing the number of threads from one to seven, reaching an average throughput of approximately 144 frames per second.

**Table 1:** Mean absolute errors of the least squares (LS) solution, adaptive unscented Kalman smoothing (AUKS) solution, and their difference, compared to the optical motion capture LS solution

Coordinate name$^{\textrm{a}}$	LS	AUKS	Difference	*p*	95% CI
Angular position (deg)					
hip_flexion_r	$36.8 \pm 11.8$	$36.6 \pm 11.7$	$0.3 \pm 1.0$	0.0020	$\left[ +0.1, \, +0.5\right]$
hip_adduction_r	$10.5 \pm 5.4$	$10.3 \pm 5.4$	$0.2 \pm 0.7$	0.0005	$\left[ +0.1, \, +0.3\right]$
hip_rotation_r	$9.2 \pm 5.4$	$10.0 \pm 5.5$	$-0.7 \pm 1.4$	0.0000	$\left[ -1.0, \, -0.5\right]$
hip_flexion_l	$36.1 \pm 12.1$	$36.0 \pm 12.1$	$0.1 \pm 0.6$	0.0785	$\left[ -0.0, \, +0.2\right]$
hip_adduction_l	$9.7 \pm 5.3$	$9.6 \pm 5.2$	$0.1 \pm 0.8$	0.1339	$\left[ -0.0, \, +0.2\right]$
hip_rotation_l	$12.4 \pm 6.9$	$12.7 \pm 6.8$	$-0.3 \pm 1.1$	0.0089	$\left[ -0.5, \, -0.1\right]$
knee_angle_r	$12.5 \pm 6.4$	$12.3 \pm 6.5$	$0.2 \pm 1.5$	0.1992	$\left[ -0.1, \, +0.5\right]$
knee_angle_l	$11.5 \pm 5.7$	$11.2 \pm 5.7$	$0.2 \pm 1.0$	0.0091	$\left[ +0.1, \, +0.4\right]$
knee_add_r	$5.9 \pm 2.6$	$6.1 \pm 2.2$	$-0.2 \pm 1.4$	0.1555	$\left[ -0.4, \, +0.1\right]$
knee_add_l	$6.3 \pm 2.3$	$6.7 \pm 2.2$	$-0.4 \pm 0.8$	0.0000	$\left[ -0.5, \, -0.3\right]$
ankle_angle_r	$10.6 \pm 4.6$	$10.9 \pm 4.8$	$-0.4 \pm 0.7$	0.0000	$\left[ -0.5, \, -0.2\right]$
ankle_angle_l	$7.4 \pm 4.5$	$7.5 \pm 4.4$	$-0.1 \pm 0.4$	0.0012	$\left[ -0.2, \, -0.1\right]$
Angular velocity (deg/s)					
hip_flexion_r	$40.7 \pm 17.6$	$40.5 \pm 17.4$	$0.3 \pm 2.4$	0.2452	$\left[ -0.2, \, +0.7\right]$
hip_adduction_r	$37.9 \pm 12.0$	$36.5 \pm 11.2$	$1.4 \pm 4.8$	0.0012	$\left[ +0.6, \, +2.3\right]$
hip_rotation_r	$32.7 \pm 16.8$	$34.6 \pm 17.1$	$-1.9 \pm 6.5$	0.0013	$\left[ -3.1, \, -0.8\right]$
hip_flexion_l	$39.2 \pm 18.7$	$38.9 \pm 18.8$	$0.3 \pm 1.6$	0.0283	$\left[ +0.0, \, +0.6\right]$
hip_adduction_l	$35.7 \pm 12.4$	$34.3 \pm 12.4$	$1.4 \pm 3.4$	0.0000	$\left[ +0.8, \, +2.0\right]$
hip_rotation_l	$34.8 \pm 17.3$	$35.0 \pm 16.8$	$-0.3 \pm 4.4$	0.5200	$\left[ -1.0, \, +0.5\right]$
knee_angle_r	$32.6 \pm 20.5$	$30.8 \pm 19.6$	$1.7 \pm 2.8$	0.0000	$\left[ +1.2, \, +2.2\right]$
knee_angle_l	$30.8 \pm 18.3$	$29.3 \pm 18.7$	$1.5 \pm 2.8$	0.0000	$\left[ +1.0, \, +2.0\right]$
knee_add_r	$31.0 \pm 16.7$	$32.7 \pm 11.9$	$-1.7 \pm 12.9$	0.1566	$\left[ -4.0, \, +0.6\right]$
knee_add_l	$34.6 \pm 14.2$	$37.5 \pm 14.5$	$-2.9 \pm 6.1$	0.0000	$\left[ -4.0, \, -1.8\right]$
ankle_angle_r	$29.4 \pm 12.6$	$30.7 \pm 14.0$	$-1.3 \pm 4.8$	0.0047	$\left[ -2.1, \, -0.4\right]$
ankle_angle_l	$33.8 \pm 16.4$	$31.2 \pm 15.8$	$2.6 \pm 3.4$	0.0000	$\left[ +2.0, \, +3.2\right]$
Angular acceleration (deg/s$^{2}$)					
hip_flexion_r	$586.1 \pm 217.3$	$567.4 \pm 193.0$	$18.7 \pm 44.6$	0.0000	$\left[ +10.7, \, +26.6\right]$
hip_adduction_r	$513.5 \pm 185.1$	$475.3 \pm 178.0$	$38.2 \pm 48.6$	0.0000	$\left[ +29.5, \, +46.9\right]$
hip_rotation_r	$653.8 \pm 356.6$	$645.5 \pm 331.3$	$8.4 \pm 102.1$	0.3677	$\left[ -9.9, \, +26.6\right]$
hip_flexion_l	$600.9 \pm 233.5$	$580.3 \pm 226.4$	$20.6 \pm 41.2$	0.0000	$\left[ +13.2, \, +27.9\right]$
hip_adduction_l	$513.7 \pm 232.0$	$471.6 \pm 210.8$	$42.1 \pm 63.7$	0.0000	$\left[ +30.7, \, +53.6\right]$
hip_rotation_l	$641.8 \pm 338.3$	$622.2 \pm 327.4$	$19.6 \pm 80.5$	0.0081	$\left[ +5.2, \, +34.1\right]$
knee_angle_r	$465.7 \pm 275.1$	$463.8 \pm 250.6$	$1.8 \pm 67.5$	0.7660	$\left[ -10.3, \, +13.9\right]$
knee_angle_l	$501.6 \pm 255.8$	$486.5 \pm 247.2$	$15.2 \pm 57.8$	0.0044	$\left[ +4.8, \, +25.5\right]$
knee_add_r	$502.2 \pm 211.4$	$434.7 \pm 159.6$	$67.5 \pm 192.9$	0.0002	$\left[ +32.9, \, +102.0\right]$
knee_add_l	$504.7 \pm 223.2$	$503.4 \pm 216.6$	$1.3 \pm 87.9$	0.8754	$\left[ -14.5, \, +17.0\right]$
ankle_angle_r	$565.3 \pm 238.0$	$563.9 \pm 241.9$	$1.4 \pm 91.3$	0.8660	$\left[ -15.0, \, +17.8\right]$
ankle_angle_l	$671.7 \pm 337.5$	$579.6 \pm 311.8$	$92.1 \pm 73.9$	0.0000	$\left[ +78.9, \, +105.4\right]$

$^{\textrm{a}}$ Names are presented as they appear in the musculoskeletal model

Results expressed as mean ± standard deviation

95% CI–95% confidence interval around the difference in population means

**Table 2:** Results of the throughput test

Number of threads in the threadpool	Throughput (1/s)$^{\textrm{a}}$
1	$53.79 \pm 1.61$
3	$90.42 \pm 0.92$
7	$144.15 \pm 1.37$

$^{\textrm{a}}$ Expressed as mean ± standard deviation over 5 runs

## Discussion

Based on the MAEs (Table 1), IMU-driven kinematics estimated with AUKSMIKT are more similar than the LS solutions to the gold standard—the LS solutions based on optical motion capture data. The relatively large differences in angular positions are partially explained by the differences in the model calibration; for each trial, the MS model was recalibrated using the first data frame of the trial, while optical motion capture data used the same calibration in all trials for the subject. This, compounded with the artifacts caused by soft tissue movement [39, 40], possible errors in IMU orientation measurements due to magnetic interference [41], and the difference between the assumed calibration pose of a test subject [42] and the default pose of the MS model in which the body segments are vertically aligned, makes the direct comparison of angular positions challenging.

IMU-driven kinematics have also been reconstructed with machine learning methods. In [43], a deep convolutional recurrent neural network was trained to map accelerometer and gyroscope readings from 5 IMUs to joint angular positions obtained from optical motion capture, resulting in average MAEs between 2.0 and 4.8 degrees for lower-body kinematics for test subjects walking at a speed of 6 km/h. In [44], a bi-directional recurrent neural network was trained to utilize accelerometer readings in addition to orientation data from only 6 IMUs to reconstruct human whole-body poses, resulting in mean joint angular position errors ranging from 15.77 to 17.54 degrees. In [45], both convolutional and long short-term memory (LSTM) networks were investigated to map accelerometer and gyroscope readings, in addition to the orientation data, from 8 IMUs to reconstruct lower-body kinematics of walking subjects. LSTM networks trained on a relatively small (11 subjects) dataset resulted in over 100% larger root-mean-square errors for hip and knee angles compared to the LS solution of OpenSim, but it was demonstrated that these errors could be reduced to an almost similar level by fine-tuning the networks with subject-specific transfer learning [45]. In [46], a random forest regressor was trained to map accelerometer and gyroscope readings from 7 IMUs to joint angular positions obtained from optical motion capture, resulting in average root-mean-square errors ranging from 2.95 to 8.32 degrees for lower-body kinematics. Besides the results presented in [43] which are considerably closer to the reported optical motion capture results, the above-mentioned methods have achieved comparable levels of accuracy compared to AUKSMIKT for angular positions of knee and ankle joints (Table 1).

For given models (1) and (2), using correct statistics for process noise $\textbf{w}$ and observation noise $\textbf{v}$ is very important for the UKF to converge to a relevant solution [20]. Observation noise may be assumed to be limited in magnitude and to change relatively slowly in IMUs [47], but the process noise may change relatively quickly in the context of human motion tracking; for example, the subject could switch from standing still to sprinting within a second. Therefore, one would prefer to use relatively high values for $\eta _{\textbf{w}}$ and relatively low values for $\eta _{\textbf{v}}$, as we have done in this work.

Mutual independence between process noise components that correspond to different coordinates is a potential limitation in this work. Cross-correlations of kinematics between different coordinates are evident in cyclic motions such as walking [48–50], which could be exploited in the process noise covariance update step. We found that updating the process noise exactly as described by lines 18–19 in Algorithm 2, however, often led to numerical instabilities due to non-positive definite covariance matrices $\textbf{P}_{(k)}^{-}$, so we opted to update the process noise as described by equations (10) – (14). A possible expansion to our modeling choice could involve updating the intra-coordinate process noise covariance components using equations (10) – (14), and updating inter-coordinate components using line 19 of Algorithm 2 if those coordinates are sufficiently spatially close to each other as defined by the topology of the MS model. That is, for example, the coordinates of the right hip would be allowed to correlate with themselves, with the coordinates of the right knee, the coordinates of the left hip, and the coordinates of the back.

Based on the throughput test, the performance of AUKSMIKT is sufficiently high for potential real-time processing on a mid-range laptop computer. Naturally, the throughput decreases as the number of IMUs and/or the length of the state vector $\textbf{x}_{(k)}$ increases, which is the case when the motion of the human upper body is tracked in addition to the lower body; however, a larger number of IMUs may also require using a lower sampling frequency due to limited bandwidth, in which case the lower throughput may still be sufficient. In addition, a CPU with a higher number of cores will also alleviate this challenge—one of the most time-consuming steps in the execution of UKF is propagating the sigma points through the observation model (Algorithm 2, line 10), which fortunately can be parallelized. In [51], full-body kinematics was solved in 0.8 ms per time frame from optical motion capture data using an EKF algorithm; their solution used the Eigen template library which was further configured to utilize Intel Math Kernel Library (MKL) optimizations. Our UKF-based tool also performs the update of noise statistics at each time frame, which further increases the processing time.

It is worth noting that the UKF implementation presented in this work is the *non-augmented* form. In the *augmented* UKF [19], the state estimation problem is formulated using augmented state means

$$ \bar{\textbf{x}}_{(k)}^{\text {a}} = \begin{bmatrix}\bar{\textbf{x}}_{(k)}^{T}&\bar{\textbf{w}}_{(k)}^{T}&\bar{\textbf{v}}_{(k)}^{T}\end{bmatrix}^{T} $$

and augmented state covariances

$$ \bar{\textbf{P}}_{(k)}^{\text {a}} = \begin{bmatrix} \textbf{P}_{(k)} &  \textbf{0} &  \textbf{0} \\ \textbf{0} &  \textbf{Q}_{(k)} &  \textbf{0} \\ \textbf{0} &  \textbf{0} &  \textbf{R}_{(k)} \end{bmatrix}. $$

In this case, $2(n + n + m) + 1$ sigma points are sampled from the distribution of $\textbf{x}_{(k)}^{\text {a}}$ instead of $2n + 1$ sigma points from the distribution of $\textbf{x}_{(k)}$. The augmented UKF may produce more accurate estimates of the state [52], but is computationally more expensive due to a larger number of sigma points. However, in our case, taking advantage of the linearity of the process model (3) enables skipping the unscented transform in the prediction phase (Algorithm 2, lines 1–7) and using the augmented form only in the update phase (Algorithm 2, lines 8–21), which requires sampling and propagating only $2(n + m) + 1$ sigma points. In our experiments, the augmented form of AUKSMIKT has demonstrated decreased numerical stability, resulting in non-positive definite covariance matrices and diverging results. Therefore, we opted to use the value $3 - n$ for the hyperparameter $\kappa$ in non-augmented UKF, since this value leads to estimates which are equivalent to those of the augmented UKF with $\kappa = 3 - (n+m)$ [52], as well as capturing higher-order moment information of the state distribution [53, 54]. Similar to Table 1, Table 3 in Appendix 8 shows a comparison of MAEs for the augmented and non-augmented forms of AUKSMIKT conducted over 127 gait trials from the Kuopio Gait dataset.

In this work, we used a process model that considered time derivatives of angular positions up to the 2nd order. Based on our preliminary experiments, inclusion of higher-order time derivatives did not improve the accuracy of AUKSMIKT. In addition, since inclusion of higher orders leads to larger sizes of state covariance matrices $\textbf{P}_{(k)}$, the computation time increases due to a larger number of sigma points to be propagated in the UKF algorithm.

The choice of lag *L* in the fixed-lag RTS smoother is a compromise between accuracy of the solution and feasibility in potential real-time applications. Real-time applications require updating the estimate for body pose as soon as possible. Thus, even the relatively modest lag of 10 samples used in this work, which corresponds to 100 ms with a 100 Hz sampling frequency, may be considered too large for, e.g., real-time feedback software [55], especially with additional delays associated with online data streaming; conversely, even a large lag of 140 ms can be considered reasonable [56] in certain application contexts. Nevertheless, we suggest using at least a short lag to counteract the delays of angular velocities and accelerations with respect to the angular positions, which can be observed if no RTS smoothing is used [9].

In multi-stage human motion analysis, uncertainties of the estimated kinematics can significantly affect the subsequent estimates of kinetics (moments and forces) [57, 58]; for example, it has been shown that a peak ankle plantarflexion angle variation of 15.9 degrees can vary the plantarflexion moment by as much as 26.6 N$\cdot$m [59]. In addition, the estimates of kinetics are further affected by the uncertainties of the body mass distribution (scaling of dimensions of body segments included) of the test subject; for example, a 1.7-kg reduction of thigh mass for a 72.6-kg man can change the knee flexion moment by approximately 5% over a gait cycle [60]. AUKSMIKT provides estimates for the uncertainties of kinematics due to the IMU observation noise and the underlying process model (Figures 1 and 2), which can be incorporated into the next stage of human motion analysis—Bayesian estimation of moments and forces.

In conclusion, we developed an extension to the OpenSim software to obtain probability distributions of human full-body kinematics from inertial motion capture data based on the adaptive unscented Kalman filter and fixed-lag RTS smoother. This extension

- can estimate the mean values of kinematics with a higher accuracy (up to 13.7% lower mean absolute errors in angular accelerations of body joints) than the native OpenSim inverse kinematics tool,
- provides estimates for the uncertainties of kinematics as covariance matrices, which can further be utilized in probabilistic estimation of full-body kinetics, and
- can process the IMU data with a sufficiently high throughput for real-time applications.

In addition, the source code for this extension was made publicly available. This enables OpenSim users to incorporate these methods into their own studies and benefits the entire biomechanics community.

## Data Availability

Not applicable. Not applicable. The source code for OpenSim extended with our custom-created tools is available on GitHub at https://github.com/Sandmaenchen/opensim-core-public/tree/ukf-uks-tools.

## Implementation of AUKSMIKT

We implemented AUKSMIKT as a C++ class extending the API of the OpenSim software (v4.5) [5]. This implementation was built to supplement the code available on GitHub in the *main* branch of OpenSim [61]. AUKSMIKT is a customized version of the native IMU-driven inverse kinematics tool of OpenSim, and it partially utilizes the source code from the Kalman smoothing algorithm implementation of De Groote et al. [9, 62] to properly benefit from the Simbody physics engine utilized by OpenSim. In addition, AUKSMIKT utilizes the C++ template library Eigen (v3.3.7) for linear algebra and quaternion algebra computations. The source code of our implementation is available on GitHub (https://github.com/Sandmaenchen/opensim-core-public/tree/ukf-uks-tools), and is released under the Apache License 2.0.

AUKSMIKT scans the musculoskeletal (MS) model file for generalized coordinate variables (angular positions of the joints and a translational position for a reference body, e.g., the pelvis) and the data file for observed IMUs to choose correct sizes for state and observation vectors and covariance matrices. The coordinates that are defined as either locked (non-changeable), translational, or dependent on other coordinates, are ignored and not included in the estimated state vector. As a consequence, our solution uses mappings between the elements of the state vector in Eigen and the state vector in OpenSim/Simbody. Most of the computationally expensive linear algebra and quaternion algebra computations are performed using vector and matrix objects of Eigen, but computations that can be performed exclusively with features from OpenSim/Simbody are computed with objects of OpenSim/Simbody, and the numeric outputs are transmitted between objects of these libraries as needed by using the map container from the C++ standard library.

To parallelize execution, the main function in AUKSMIKT spawns two threads: thread A to run the $\textit{updateAUKF}(\cdot )$ function, and thread B to run the $\textit{backwardSmooth}(\cdot )$ function and write the elements of $\bar{\textbf{x}}_{(k)}^{\text {smooth}}$ and $\textbf{P}_{(k)}^{\text {smooth}}$ to their resultant text files. Furthermore, thread A uses a custom-made thread pool class to enable parallel execution by multiple CPU cores in the parts of the $\textit{updateAUKF}(\cdot )$ code where this is feasible (e.g., line 10 in Algorithm 2). The source code for this class was obtained by prompting the free version of the online ChatGPT chatbot (Model GPT$-$3.5; OpenAI, USA) with a phrase, “Can you write a C++ example that uses thread pool to process, in parallel, columns of a matrix of Eigen library,” and afterward specifying that we are working with C++11. We edited the resulting source code to prevent the parent thread from proceeding while at least one thread in the thread pool is still working.

For better numerical stability, AUKSMIKT constrains the $\sigma _{i}^{2}$ values during noise update using user-defined lower and upper limits $\sigma _{\text {min}}^{2}$ and $\sigma _{\text {max}}^{2}$, respectively. The idea is to keep the magnitude above $\sigma _{\text {min}}^{2}$ for each coordinate *i* to allow the UKF to scan the neighborhood of $\bar{\textbf{x}}_{(k)}$ from sufficiently far away, to quickly adapt to large changes after a kinematically stable time period. Likewise, limiting each coordinate to values smaller than $\sigma _{\text {max}}^{2}$ prevents the solution from diverging due to sudden, very large changes in kinematics, possibly caused by an artifact in the observations.

Based on the user’s choice, AUKSMIKT can conform to inequality constraints, which in OpenSim are defined as the *clamped* property of the generalized coordinates. This has been implemented by “clipping” the state means $\bar{\textbf{x}}_{(k)}$, $\bar{\textbf{x}}_{(k)}^{-}$, and $\bar{\textbf{x}}_{(k)}^{\text {smooth}}$ back to the window of permitted values if their elements violate these constraints [63]. In practice, if the angular position of coordinate *i* is outside of the window of permitted values, it is set equal to the closest boundary value, and the corresponding higher-order derivatives are set to zero if they are positive/negative for the coordinate with too high/low angular position value, respectively.

Similar to the native IMU-driven inverse kinematics tool, AUKSMIKT requires as inputs the file containing the OpenSim MS model, the file containing the time series of IMU orientations as quaternions, a vector containing three Euler angles (rotation sequence XYZ) to align the ground coordinate axes of the IMUs with those of OpenSim, a directory to store the results, and the time window over which the inverse kinematics are to be computed. In addition, the user needs to set

- the highest order *D* of time derivatives of angular positions to include in the state vectors $\textbf{x}_{(k)}$,
- the length of lag *L* to use in the fixed-lag RTS smoothing,
- the number of threads to be used in the thread pool (integer; if 0, this number is set equal to the number of machine cores),
- the hyperparameters $\alpha$, $\beta$, and $\kappa$ (value $\kappa = -1.337$ sets $\kappa = 3-n$),
- the initial value to be used for coefficients $\sigma _{i}^{2}$ to construct the initial covariance matrix $\textbf{Q}_{(1)}$,
- the lower and upper limits $\sigma _{\text {min}}^{2}$ and $\sigma _{\text {max}}^{2}$ for updating the covariance matrix $\textbf{Q}_{(k)}$,
- the RMS error values for roll, heading and pitch of the IMUs to set to construct the initial covariance matrix $\textbf{R}_{(1)}$,
- the scalar values $c_i,\, i = 0,1,\dots ,D$ (all equal to one by default),
- the forgetting factors $\eta _{\textbf{w}}$ and $\eta _{\textbf{v}}$ to be used in noise estimation, and
- whether to conform the solution to inequality constraints, if there are any.

Optionally, the user can also enforce that the observation noise $\textbf{v}_{(k)}$ is modeled as zero-mean, that the IMUs are *not* mutually independent, and that the process noise update does *not* follow the steps outlined in (10) – (14).

AUKSMIKT outputs a file containing the time series of angular position for each generalized coordinate of the MS model, which can afterward be visualized in the OpenSim graphical user interface with the MS model. In addition, AUKSMIKT optionally provides two files—one file contains the estimated means $\bar{\textbf{x}}_{(k)}^{\text {smooth}}$ and the other contains the estimated covariances $\textbf{P}_{(k)}^{\text {smooth}}$ of the state.

## Preparation of Data

The raw IMU sensor data subsets were provided as series of MATLAB structures in the original format. We extracted the sensor values into standard text files. The sampling rate information (100 Hz) was added to the header of each output file so that the data reader could create a time range that matches that of the optical motion capture data.

The OpenSim API class *XsensDataReader* was used to map the individual sensor readings to the proper body segments (e.g., left shank, pelvis, etc.) of the MS model. The correspondence of IMUs to body segments was obtained from the description of the dataset. During processing with this utility, the IMU orientation data were converted from their original representation as rotation matrices to quaternions for further use in OpenSim.

The OpenSim API class *C3DFileAdapter* was used to extract the optical marker data from the original Vicon ‘.trc’ files. These data were used to compute the LS solution for the inverse kinematics problem based on optical motion capture. Following the extraction of the optical motion capture data, subject-specific calibration was performed using the OpenSim API class *ScaleTool*.

The calibration step scales the body segments of the base skeleton using a combination of manual measurements and calibration pose experimental marker measurements. The manually measured inter-asis distance (IAD) and the lengths of left and right femur and shank were used to scale the respective segments by modifying the *ScaleTool* class to accept manual distance measurements. The foot and torso were scaled based on the difference between the model marker set and the experimental calibration pose marker set. Following the subject-specific scaling, the model optical markers were refitted onto the resulting skeleton to closely match the experimental markers for the specific participant in the calibration pose.

## Optical Motion Capture Solutions

Since the native OpenSim inverse kinematics tool for optical motion capture data failed when too few markers on the walking subject could be seen by the cameras (marker locations recorded as not-a-number), we increased the starting time from the beginning of the trial (0 s) by 0.5-s increments until the solution could be obtained. Likewise, if the solution failed toward the end of the trial due to the subject walking past the FOV of the cameras, the ending time of the trial was reduced by 0.5-s steps until the solution could be obtained. Since some of these solutions still contained unrealistic poses (such as the lumbar being extended by more than 40 degrees backwards) at the beginning or the end of the trial, these solutions were truncated by 0.5 s from both the beginning and the end. The final durations of these truncated solutions varied between 1.5 s and 4.0 s (mean: 2.83 s, standard deviation: 0.75 s).

## Comparison of Augmented and Non-augmented AUKS Solutions

We compared the performance of augmented form of AUKSMIKT to the performance of the non-augmented form of AUKSMIKT presented in this work. The augmented form of this tool had the same settings as the non-augmented form (described in Sect. 2.3.2), with the exception that we set $\kappa = 0$ for better numerical stability. Similar to the test carried out in Sects. 2.3.2–2.3.3, we computed the mean absolute errors (MAEs) for both augmented and non-augmented adaptive unscented Kalman smoothing solutions over 127 gait trials from the Kuopio Gait dataset (two trials from subject ‘03’ could not be processed and the solution of the augmented form diverged in one trial from subject ‘08’) and evaluated the statistical significance of their differences. The results are presented in Table 3.

**Table 3:** Mean absolute errors of the augmented adaptive unscented Kalman smoothing (A-AUKS) solution, non-augmented adaptive unscented Kalman smoothing (N-AUKS) solution, and their differences, compared to the optical motion capture LS solution

Coordinate name$^{\textrm{a}}$	A-AUKS	N-AUKS	Difference	*p*	95% CI
Angular Position (deg)					
hip_flexion_r	$37.3 \pm 12.2$	$36.7 \pm 12.0$	$\phantom {-}0.5 \pm 1.3$	0.0000	$\left[ +0.3, \, +0.8\right]$
hip_adduction_r	$10.0 \pm 5.4$	$10.3 \pm 5.5$	$-0.2 \pm 0.6$	0.0000	$\left[ -0.3, \, -0.1\right]$
hip_rotation_r	$9.8 \pm 5.4$	$10.0 \pm 5.6$	$-0.2 \pm 1.4$	0.0511	$\left[ -0.5, \, +0.0\right]$
hip_flexion_l	$36.1 \pm 12.3$	$36.1 \pm 12.3$	$-0.0 \pm 1.4$	0.9951	$\left[ -0.3, \, +0.3\right]$
hip_adduction_l	$9.7 \pm 5.3$	$9.6 \pm 5.3$	$\phantom {-}0.0 \pm 0.4$	0.3943	$\left[ -0.0, \, +0.1\right]$
hip_rotation_l	$13.1 \pm 7.0$	$12.8 \pm 6.9$	$\phantom {-}0.3 \pm 1.0$	0.0004	$\left[ +0.1, \, +0.5\right]$
knee_angle_r	$12.6 \pm 6.3$	$12.3 \pm 6.4$	$\phantom {-}0.3 \pm 1.5$	0.0486	$\left[ +0.0, \, +0.5\right]$
knee_angle_l	$11.4 \pm 5.6$	$11.2 \pm 5.6$	$\phantom {-}0.1 \pm 0.9$	0.0586	$\left[ -0.0, \, +0.3\right]$
knee_add_r	$6.7 \pm 2.9$	$6.1 \pm 2.1$	$\phantom {-}0.6 \pm 1.5$	0.0000	$\left[ +0.3, \, +0.9\right]$
knee_add_l	$6.6 \pm 2.3$	$6.7 \pm 2.2$	$-0.2 \pm 0.7$	0.0025	$\left[ -0.3, \, -0.1\right]$
ankle_angle_r	$11.8 \pm 5.4$	$10.9 \pm 4.8$	$\phantom {-}0.9 \pm 1.7$	0.0000	$\left[ +0.6, \, +1.2\right]$
ankle_angle_l	$7.5 \pm 4.3$	$7.6 \pm 4.5$	$-0.0 \pm 0.6$	0.4083	$\left[ -0.2, \, +0.1\right]$
Angular Velocity (deg/s)					
hip_flexion_r	$41.6 \pm 17.3$	$41.0 \pm 17.4$	$\phantom {-}0.7 \pm 3.3$	0.0223	$\left[ +0.1, \, +1.2\right]$
hip_adduction_r	$35.8 \pm 11.2$	$36.8 \pm 11.2$	$-1.0 \pm 2.1$	0.0000	$\left[ -1.3, \, -0.6\right]$
hip_rotation_r	$34.6 \pm 17.4$	$35.0 \pm 17.3$	$-0.4 \pm 6.7$	0.5078	$\left[ -1.6, \, +0.8\right]$
hip_flexion_l	$39.3 \pm 18.7$	$39.3 \pm 18.7$	$\phantom {-}0.0 \pm 2.6$	0.8858	$\left[ -0.4, \, +0.5\right]$
hip_adduction_l	$34.8 \pm 12.4$	$34.4 \pm 12.3$	$\phantom {-}0.4 \pm 1.5$	0.0052	$\left[ +0.1, \, +0.6\right]$
hip_rotation_l	$34.3 \pm 16.0$	$35.3 \pm 16.7$	$-1.1 \pm 3.1$	0.0002	$\left[ -1.6, \, -0.5\right]$
knee_angle_r	$33.2 \pm 20.5$	$31.5 \pm 19.9$	$\phantom {-}1.7 \pm 3.3$	0.0000	$\left[ +1.2, \, +2.3\right]$
knee_angle_l	$29.6 \pm 18.4$	$29.7 \pm 19.0$	$-0.1 \pm 2.2$	0.6115	$\left[ -0.5, \, +0.3\right]$
knee_add_r	$35.3 \pm 17.0$	$33.0 \pm 11.8$	$\phantom {-}2.4 \pm 10.6$	0.0138	$\left[ +0.5, \, +4.2\right]$
knee_add_l	$35.8 \pm 15.9$	$37.9 \pm 14.7$	$-2.1 \pm 5.2$	0.0000	$\left[ -3.0, \, -1.1\right]$
ankle_angle_r	$37.6 \pm 19.3$	$31.0 \pm 14.0$	$\phantom {-}6.7 \pm 10.2$	0.0000	$\left[ +4.9, \, +8.5\right]$
ankle_angle_l	$32.0 \pm 16.3$	$31.7 \pm 16.5$	$\phantom {-}0.4 \pm 3.0$	0.1712	$\left[ -0.2, \, +0.9\right]$
Angular Acceleration (deg/s$^{2}$)					
hip_flexion_r	$586.2 \pm 199.6$	$574.7 \pm 194.4$	$\phantom {-}11.5 \pm 66.9$	0.0558	$\left[ -0.3, \, +23.3\right]$
hip_adduction_r	$478.4 \pm 184.9$	$479.7 \pm 178.4$	$-1.3 \pm 30.5$	0.6275	$\left[ -6.7, \, +4.1\right]$
hip_rotation_r	$642.9 \pm 349.1$	$653.5 \pm 332.8$	$-10.5 \pm 77.9$	0.1322	$\left[ -24.3, \, +3.2\right]$
hip_flexion_l	$590.5 \pm 228.9$	$585.7 \pm 224.3$	$\phantom {-}4.9 \pm 46.8$	0.2441	$\left[ -3.4, \, +13.1\right]$
hip_adduction_l	$475.3 \pm 212.5$	$474.4 \pm 209.5$	$\phantom {-}0.9 \pm 23.7$	0.6655	$\left[ -3.3, \, +5.1\right]$
hip_rotation_l	$615.1 \pm 317.7$	$629.3 \pm 325.6$	$-14.2 \pm 41.4$	0.0002	$\left[ -21.5, \, -6.9\right]$
knee_angle_r	$499.6 \pm 262.9$	$473.5 \pm 256.6$	$\phantom {-}26.2 \pm 71.6$	0.0001	$\left[ +13.5, \, +38.8\right]$
knee_angle_l	$499.8 \pm 250.2$	$490.7 \pm 248.9$	$\phantom {-}9.2 \pm 38.7$	0.0090	$\left[ +2.3, \, +16.0\right]$
knee_add_r	$450.3 \pm 171.2$	$437.6 \pm 159.3$	$\phantom {-}12.8 \pm 139.0$	0.3041	$\left[ -11.7, \, +37.3\right]$
knee_add_l	$485.6 \pm 242.7$	$507.1 \pm 218.2$	$-21.6 \pm 79.8$	0.0029	$\left[ -35.6, \, -7.5\right]$
ankle_angle_r	$723.1 \pm 460.1$	$570.8 \pm 245.2$	$152.3 \pm 326.3$	0.0000	$\left[ +94.8, \, +209.8\right]$
ankle_angle_l	$590.8 \pm 323.2$	$587.3 \pm 323.5$	$\phantom {-}3.5 \pm 40.2$	0.3337	$\left[ -3.6, \, +10.6\right]$

a Names are presented as they appear in the musculoskeletal model

Results expressed as mean ± standard deviation

95% CI–95% confidence interval around the difference in population means

For A-AUKS, $\kappa = 0$, and for N-AUKS, $\kappa = 3 - n$
